# A multicenter prospective cohort study to develop frailty-based prognostic criteria in heart failure patients (FLAGSHIP): rationale and design

**DOI:** 10.1186/s12872-018-0897-y

**Published:** 2018-08-02

**Authors:** Sumio Yamada, Takuji Adachi, Hideo Izawa, Toyoaki Murohara, Takaaki Kondo, on behalf of the FLAGSHIP collaborators, Sumio Yamada, Hideo Izawa, Toyoaki Murohara, Takaaki Kondo, Horoki Matsui, Takuji Adachi, K. Iwatsu, R. Fujita, K. Kamisaka, E. Nakane, D. Sakui, I. Kawamura, K. Shibata, M. Ehara, H. Otake, T. Shimozato, T. Abe, T. Mizuno, Y. Iida, T. Yamada, T. Nagao, K. Sakamoto, T. Ando, K. Nishigaki, N. Iritani, M. Terashima, T. Ito, N. Fujimoto, T. Soga, K. Hayashi, T. Wakita, R. Ishiki, K. Kobayashi, T. Okumura, S. Uchiyama, M. Nishi, Y. Sasamoto, N. Endo, T. Hasegawa, K. Harada, N. Sato, H. Origuchi, S. Hanada, H. Iwakiri, Y. Kasahara, K. Omiya, Y. Kono, H. Izawa, M. Yagi, N. Osada, N. Takeichi, K. Kida, J. Hirasawa, T. Kanbara, Y. Ogawa, T. Ishihara, K. Kondo, F. Fukukawa, M. Uemura, K. Mizutani, Y. Tsunekawa, D. Tanimura, N. Ban, N. Tsuboi, H. Maeda, K. Kawai

**Affiliations:** 1 Department of Health Sciences, Nagoya University Graduate School of Medicine, 1-1-20 Daiko-minami, Higashi-ku, Nagoya, 461-8673 Japan; 2 Program in Physical and Occupational Therapy, Nagoya University Graduate School of Medicine, 1-1-20 Daiko-minami, Higashi-ku, Nagoya, 461-8673 Japan; 3 Department of Cardiology, Fujita Health University Banbuntane Hotokukai Hospital, 3-6-10 Otobashi, Nakagawa-ku, Nagoya, 454-8509 Japan; 4 Department of Cardiology, Nagoya University Graduate School of Medicine, 65 Tsurumai-cho, Showa-ku, Nagoya, 466-8550 Japan

**Keywords:** Heart failure, Frailty, Diagnostic criteria, Multicenter cohort study, Japan

## Abstract

**Background:**

Heart failure (HF) and frailty often co-exist, and frailty in HF results in a poor prognosis. However, in Asian populations, prognostic criteria are needed to examine the effect of frailty on HF. Therefore, we conducted a nationwide cohort study to develop frailty-based prognostic criteria in HF patients (FLAGSHIP). FLAGSHIP mainly aims to 1) develop the frailty criteria based on HF-specific outcomes, 2) propose a hypothesis of the potential mechanisms of frailty manifestations in HF, and 3) examine the effects of outpatient cardiac rehabilitation on frailty.

**Methods:**

In this prospective study, we consecutively enroll ambulatory patients admitted because of acute HF or exacerbation of HF and elderly patients admitted for acute myocardial infarction (age ≥ 70 years). They will be followed up for 2 years to assess frailty and hard clinical events. The primary endpoints of FLAGSHIP are cardiac events including cardiac mortality and HF-related readmission after discharge. Secondary endpoints are readmissions because of fracture or pneumonia and all-cause mortality. We used clinical data, including the items related to the frailty phenotype to develop diagnostic criteria for frailty and known prognostic factors of HF. Cognitive function, depression, and anorexia are also considered as potential components of frailty. As of March 2018, 2650 patients (85% was patients admitted for HF) have been registered from 30 collaborating hospitals nationwide in Japan.

**Discussion:**

FLAGSHIP provides diagnostic criteria and fundamental information on frailty manifestations to develop the best practices for the long-term management of HF. Diagnostic criteria on frailty developed by FLAGSHIP is expected to become a novel indicator for the stratification of patients at risk to functional decline after medical or surgical treatment, and in turn to contribute to the best practices in the long-term management of HF.

**Electronic supplementary material:**

The online version of this article (10.1186/s12872-018-0897-y) contains supplementary material, which is available to authorized users.

## Background

Frailty is a condition characterized by a decline in physiological reserve that is associated with an increased risk of adverse health outcomes when exposed to a stressor [1]. Frailty is considered a medical syndrome in elderly people, which can be improved or attenuated by interventions [1]. However, despite knowing the prognostic value of frailty, diagnostic criteria for frailty have not been established in Asian populations. The Cardiovascular Health Study frailty index is a widely used frailty phenotype model developed based on studies of the community-dwelling elderly, almost all of whom were Caucasian or African American [2]. Accordingly, the cut-off values for grip strength to diagnose weakness cannot be applied to Asians because of differences in physique. Recently, the Asian Working Group of Sarcopenia has proposed a new cut-off value for grip strength to diagnose decreased muscle strength in the general Asian population, which was based on an expert consensus [3]. The working group also proposed to develop new cut-off values for each item in the frailty index based on longitudinal outcome-based studies [3].

Meanwhile, there is a growing interest in frailty accompanied by heart failure (HF). We previously reported the results of a secondary analysis that suggested the possibility of frailty as a clinical marker for the management of HF patients [4], although there were several study limitations, including small sample size and confounding factors. Recent similar studies have also described a relationship between frailty and mortality [5, 6]. Yet, further robust evidence is required to establish the prognostic impact of frailty in patients with HF in Asian populations. Moreover, most previous studies have defined frailty in HF patients based on the frailty criteria developed among healthy community-dwelling elderly and not as a disease-specific outcome of HF.

In patients with HF, sarcopenia because of aging or cachexia, or both, does exist [7]. In a conceptual model, cachexia is proposed to be a wasting syndrome characterized by the loss of muscle and adipose tissue resulting from anorexia, chronic inflammation, insulin resistance, and hypogonadism [8]. Bacterial translocation caused by increased gut permeability is one of the major potential mechanisms underlying cardiac cachexia [9]. However, this hypothesis has been presented based on evidence in patients with HF and reduced ejection fractions (HFrEF). In consideration of the differences in pathology between HFrEF and HF with a preserved ejection fraction (HFpEF), frailty may manifest under a different mechanism, according to the HF subtype. Additionally, mental health problems, such as cognitive impairment and depression, may possibly augment frailty in HF patients [10]. Therefore, frailty in patients with HF should be discussed in a multidimensional framework.

The diagnostic criteria for frailty based on HF-specific outcomes are expected to serve as standard clinical indicators in HF management. Therefore, we conducted a nationwide, multi-center, prospective cohort study to develop the frailty-based prognostic criteria in heart failure patients (FLAGSHIP).

## Methods/design

### Study design

FLAGSHIP is an ongoing multi-center, prospective cohort study in Japan. The study patients are enrolled during hospitalizations for HF and are followed-up for 2 years after discharge. This study was designed based on the Strengthening the Reporting of Observational Studies in Epidemiology (STROBE) statement.

### Study objectives

The main objectives of FLAGSHIP include the following: 1) to develop the frailty criteria based on HF-specific clinical outcomes, 2) to propose a hypothesis regarding potential mechanisms of frailty manifestations in patients with HF, and 3) to examine the effect of outpatient cardiac rehabilitation on frailty in patients with HF.

### Study hospitals

**Fig. 1:**
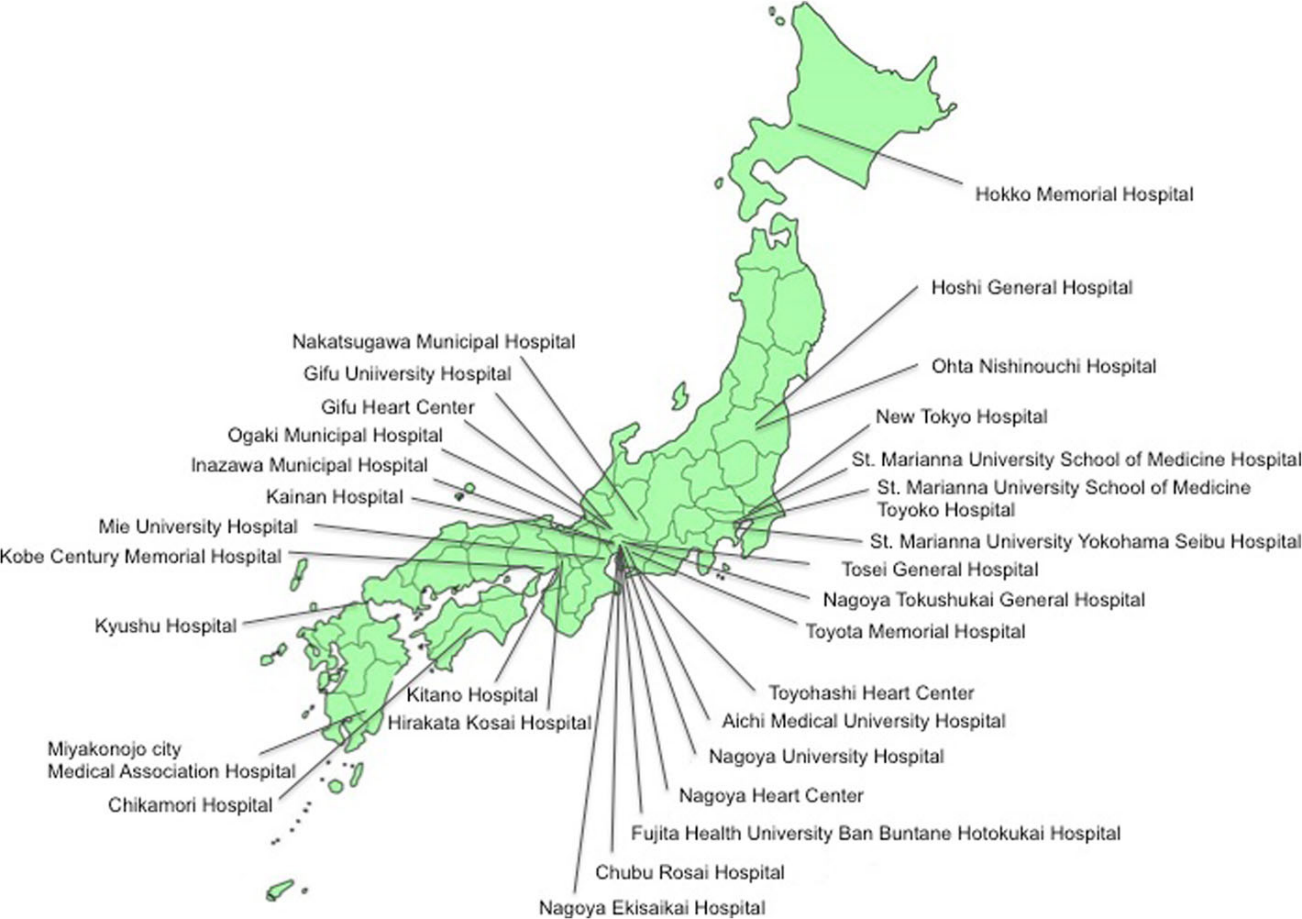
Collaborating hospitals

The participating study hospitals were selected across Japan considering their geographical distribution (Fig. 1). All the participating hospitals provided standardized cardiac inpatient rehabilitation services according to the Japanese Association of Cardiac Rehabilitation. Very few hospitals do not provide cardiac outpatient rehabilitation services.

### Study population

Eligibility criteria for FLAGSHIP include 1) ambulatory patients admitted due to HF, and 2) ambulatory patients aged 70 or over, admitted because of acute myocardial infarction (AMI). Ambulatory patients are defined as those capable of walking 20 m at the time of discharge, with or without assistance or walking aids. The exclusion criteria included the presence of one or more of the following: 1) severe cognitive impairment defined by a score on the Mini-Mental State Examination (MMSE) [11] < 17 points [12], 2) severe mental disorder, 3) difficulty in answering questionnaires, and 4) an assumed impending mortality (e.g., severe aortic valve stenosis not amenable to surgical intervention, terminal stage cancer). Patients readmitted to the hospital during the study period are enrolled at the time of the first hospitalization.

### Sample size calculation

The sample size was calculated by performing a multivariate analysis, which examined the relationship between measured frailty items and cardiac events, a primary endpoint in this study. In the multivariate analysis, 30 of the independent variables, including frailty items and known prognostic factors of HF, were selected for the analysis. Assuming 10 outcomes per one independent variable, we needed to observe 300 outcomes. Based on our preliminary data, a 20% incidence rate of a cardiac event in 2 years was estimated. With a 15% estimated drop-out rate, the necessary sample size was calculated to be 1764 patients. To perform the multivariate analysis stratified by HFrEF and HFpEF, the final target sample size was determined to be 3500.

### Data collection and processing

**Fig. 2:**
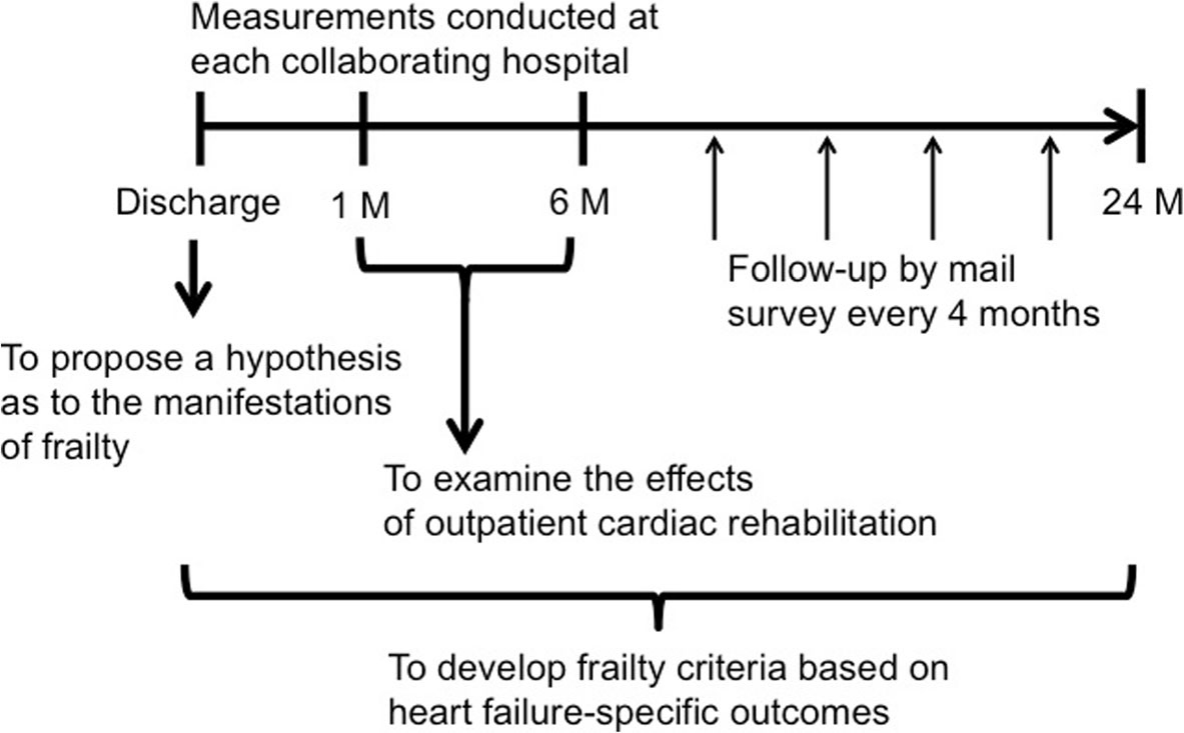
Study design to achieve the main three objectives

The study protocol is shown in Fig. 2. Development of the frailty criteria in HF, a primary objective of this study, is conducted using baseline data and subsequent follow-up surveys. Cox proportional hazards model is used to assess the independent relationship between each frailty item and study endpoints adjusted for conventional prognostic factors in HF. Then, receiver operating characteristic analysis is performed to identify a cut-off value of each frailty item to predict study endpoint. Because prognostic factors in HF are likely to differ by short-term and long-term outcomes, we assess prognostic impact of frailty on 6-month and 2-year prognosis. Study endpoints and frailty assessment are as described below.

In advance of this longitudinal analysis, a cross-sectional analysis of the baseline data is performed to examine the determinants of frailty in HFrEF and HFpEF and to propose a hypothesis for frailty manifestations in HF. Additionally, the effects of outpatient cardiac rehabilitation on frailty are examined in a non-randomized study. Any changes in the indicators of frailty from 1 to 6-months after discharge are compared between those who did and those who did not participate in cardiac rehabilitation after discharge. Propensity score matching is conducted to control for the influence of potential confounding factors.

**Fig. 3:**
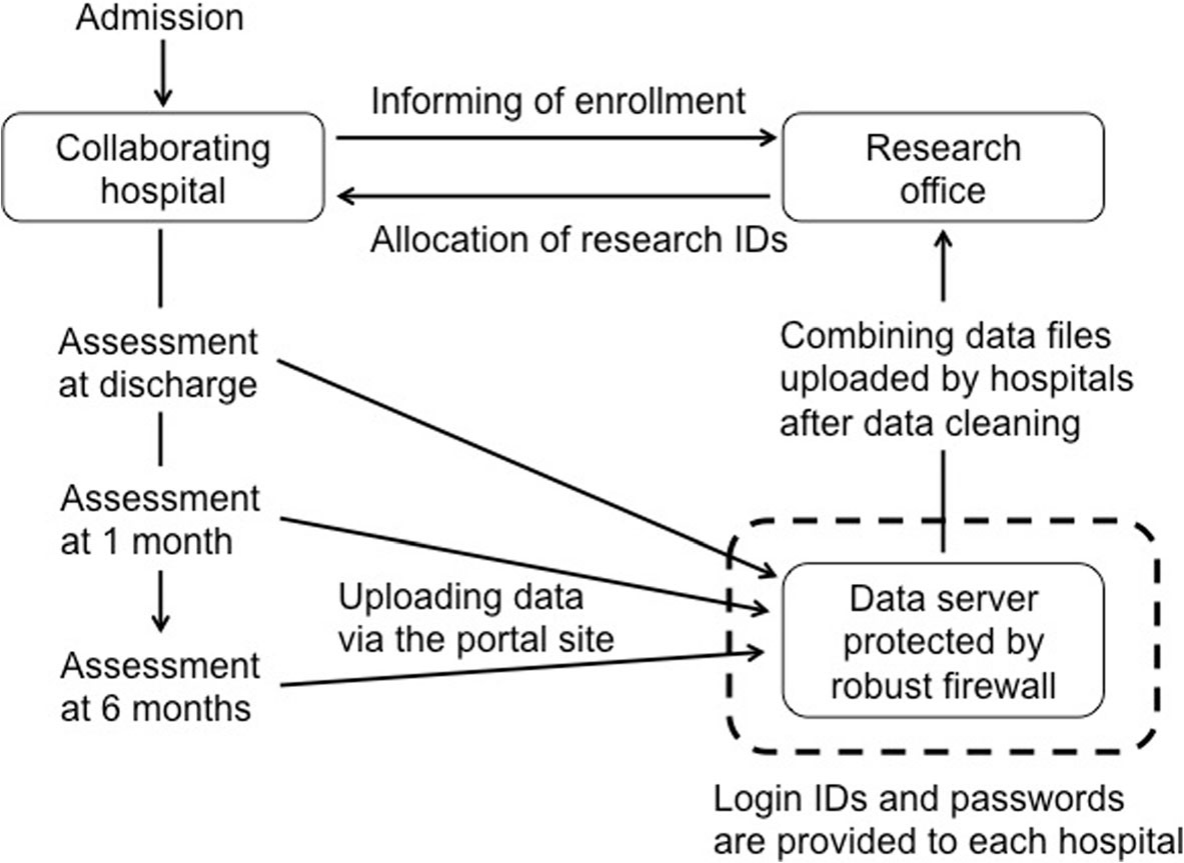
Data registration system and security for data protection

The collaborating hospitals are encouraged to register the patients as consecutively as possible. Once informed consent is obtained, the data center assigns a study ID to the patient for linkable anonymizing. Next, data are registered via the website designated for the FLAGSHIP study. For security reasons, unique IDs and passwords are assigned for each hospital to use the electronic system. The website is encrypted using secure sockets layer, and our data server is protected by a robust firewall to prevent unauthorized access to any information that we store (Fig. 3).

For each patient, the demographic data, etiologies of HF, precipitating causes, comorbidities, laboratory assessments, echocardiographic findings, medications, and frailty assessments during the hospitalization are collected. At 1 month after discharge, a frailty assessment is performed again. At 6 months, data on laboratory measurements, echocardiographic findings, medications, and frailty are obtained if the patients received outpatient treatment from the collaborating hospitals after discharge. Follow-up mail surveys are sent after 6 months for each patient, and then re-sent every 4 months until 24 months after discharge. The mail survey includes outcome surveys and frailty questionnaires. Frailty questionnaires consist of exhaustion, physical inactivity and appetite loss. Body weight at each follow-up point is also collected. Self-measurement of body weight at home is performed using the digital weight scale prepared by research office (HD-661, TANITA Corporation, Japan). Trajectories of each frailty questionnaire and body weight after discharge are analyzed using the group-based trajectory modeling as a sub-study of FLAGSHIP.

The primary endpoints of FLAGSHIP are cardiac events including cardiac mortality and HF-related readmission after discharge. Secondary endpoints are readmissions because of fracture or pneumonia and all-cause mortality.

### Frailty assessment

The frailty assessment in this study includes several aspects of frailty, in consideration of the pathophysiology of HF.

Weight loss is assessed by the body mass index (BMI). In general, unintentional weight loss in the prior 6 months is considered in the assessment of weight loss with respect to the frailty criteria. However, the mean age of Japanese patients with HF is approximately 70 years, and cognitive decline is often observed. Therefore, we selected the BMI as an objective indicator for defining weight loss instead of using self-reported assessments.

Slowness and weakness are assessed by the 10-m usual walking speed (UWS) and grip strength (GS), respectively. All measurements of grip strength are performed using the Jamar dynamometer (Digital Hand Dynamometer, DHD-1, SAEHAN Corporation, South Korea) set at the second handle position. The participants sat with the wrist in a neutral position and the elbow flexed at 90°. Before starting the enrollment of the study patients, measurement reliabilities for UWS and GS were confirmed at each hospital. The examiners, typically two to four physical therapists per hospital, measured UWS and GS twice each on five in-patients, aged approximately 70 years, on different days. Each subject was also measured by the other examiners. From that data, the intraclass and interclass correlation coefficients were calculated. Each hospital started to enroll the patients after providing > 0.9 of intraclass and interclass correlation coefficients.

Exhaustion is assessed using the Performance Measure of Activity in Daily Living-8 (PMADL-8) [13]. PMADL-8 is a questionnaire assessing functional limitations. It comprises a list of eight items potentially requiring daily physical activity in chronic heart failure by a four-category response scale. It is scored from 8 to 32, with higher scores indicating more severe functional limitations. The score is strongly and negatively correlated to the peak VO_2_ measured by the cardiopulmonary exercise test [14]. Reliability and validity of the PMADL-8 has been published elsewhere [13, 15].

Physical inactivity is assessed using a questionnaire composed of seven items with a five-point Likert scale, in consideration of clinical utility. The total score (7–35 points) has a moderate to strong correlation with step counts and moderate to vigorous physical activity and is objectively measured by an electrical accelerometer in patients after a mild stroke or with a history of cardiac disease. The validation study of this questionnaire has now been submitted.

In addition to the aforementioned physical aspects, appetite, cognitive function, and depression are also assessed as components of frailty. Appetite is assessed using Simplified Nutritional Appetite Scale (SNAQ) [16] composed of a list of four items with a four-point Likert scale, the higher scores indicate better appetites. The score of the SNAQ (4–20) predicts weight loss in the subsequent 6 months to 1 year in elderly people.

Cognitive function is assessed using an MMSE, which is a standard test to assess global cognitive function, and includes 11 questions with a maximum score of 30 [11]. Depression is assessed using a five-item geriatric depression scale (GDS5) questionnaire, and a score ≥ 2 points was defined as depression [17].

### Patient confidentiality

The study protocol of FLAGSHIP was organized to according to the Guidelines for the Epidemiological Research proposed by the Japanese Ministry of Health, Labour and Welfare. Additionally, the study protocol was approved by the ethics committee of Nagoya University School of Medicine (approval no. 2014–0421). Ethical approval was also obtained from each participating hospital (Additional file 1), and each patient provided written informed consent to be registered to this study. Treatment methods and hospital care for the patients are not altered due to participation in this study.

## Discussion

Frailty coexisting with HF is concerning in Japan as well as in Western countries because of the increasing number of patients that are living longer. To our knowledge, FLAGSHIP is the first large-scale, prospective, multicenter cohort study to develop diagnostic criteria for frailty based on HF-specific outcomes worldwide. This cohort study was also designed to propose a hypothesis regarding the potential mechanisms of frailty manifestations and to examine the effect of outpatient cardiac rehabilitation on frailty in HF. By providing these fundamental frailty data, FLAGSHIP contributes to guiding best practices for the long-term management of HF.

The prognostic impact of frailty on HF has recently been documented in several publications [4–6, 18]. We have also published the results of the secondary analysis regarding the relationship between frailty and the increased risk of cardiac events and mortality in HF [4]. However, diagnostic criteria for frailty, including the appropriate frailty items and the respective cut-off values to predict disease-specific outcomes, remain to be established. To this end, we conducted this nationwide, prospective cohort study. In this study, several aspects of the fragile state of patients were assessed. We observed physical function, appetite, and psychophysiological function in consideration of the possible mechanisms of frailty manifestations in HF. An appropriate set of frailty items and their cut-off values will be determined in the future using disease-specific outcomes and follow-up data. A trajectory analysis will also facilitate the analysis of post-discharge time-related trends in body weight and functional limitations, as well as appetite loss.

In HF, cachexia, a wasting syndrome characterized by the loss of muscle and adipose tissue [8], causes secondary sarcopenia [7], and in turn frailty. Anorexia is one of the main causes of cachexia [8], and results from intestinal ischemia or congestion, and systemic inflammation [19]. Hence, evaluating anorexia may be helpful in assessing frailty due to a cachectic state, and it is considered a candidate component of HF-induced frailty. Depression that also often coexists in HF [10] is another candidate to be potentially included for defining frailty. We previously reported that depression in HF was associated with severe functional limitations after discharge [20]. Additionally, a previous meta-analysis of the community-dwelling elderly demonstrated a bilateral relationship between depression and frailty [21]. Based on these findings, we included appetite and psychophysiological function in the frailty assessment, in addition to physical components proposed as part of the frailty phenotype.

FLAGSHIP also included patients aged ≥70 years admitted because of AMI even if they did not manifest HF. Older age is one of the major risk factors for HF [22], which may relate to frailty or mortality following AMI. Preventive management of HF, therefore, should be considered for the clinical management of the elderly with AMI. Walking speed, a major phenotype of frailty, has been reported to predict cardiovascular events after AMI [23]. In addition, a recent cohort study demonstrated a relationship between geriatric condition and poor prognosis following acute coronary syndrome [24]. From these evidences, FLAGSHIP also aimed to examine the prevalence of frailty and its trajectory in aged AMI.

Each patient enrolled into this study will be followed-up for 2 years after discharge. In our previous report [4],36 out of 181 patients (19.9%) with a mean age of 68.1 ± 9.7 years (who were younger than the patients generally seen in actual clinical practice), experienced cardiac death or a HF readmission during the 2 years after discharge. Additionally, another of our previous studies in the elderly with care needs demonstrated that > 25% of the participants experienced disability progression during the 3 years observation [25]. Based on these preliminary observations, we determined that 2 years of follow-up for FLAGSHIP was sufficient to observe study outcomes. In addition to the disease-specific outcome, readmissions due to fracture or pneumonia were frequently observed events in the fragile elderly, and are also considered to be notable outcomes in FLAGSHIP. It is easily assumed that deconditioning after fracture or pneumonia will result in the progression of frailty. In addition, systemic inflammation due to pneumonia or fracture is likely to make HF management more difficult [26, 27]. FLAGSHIP is therefore designed to examine frailty-induced clinical outcomes. Another aim of this cohort study was to examine the effects of cardiac rehabilitation on frailty. Patients are referred for cardiac rehabilitation in daily practice to provide high-quality long-term management. Exercise programs in cardiac rehabilitation have favorable effects on HF prognosis. However, there is limited evidence of the effects of cardiac rehabilitation among HF patients with frailty or sarcopenia, because such patients are often excluded from clinical trials. Observational results with a larger study sample size will allow us to analyze the effects of cardiac rehabilitation on frailty in real-world clinical practice. Along with these, many sub-studies are currently going beside the main stream, for instance, hypothesis generation to become a fragile based on HF subtype, etc. Future reports from FLAGSHIP will continue to provide clinical information that will be of value in clinical practice.

## Conclusions

Diagnostic criteria on frailty developed by FLAGSHIP is expected to become a novel indicator for the stratification of patients at risk to functional decline after medical or surgical treatment, and in turn to contribute to the best practices in the long-term management of HF.

## Additional files

Additional file 1: Hospitals and Ethics Committees enrolled in the FLAGSHIP study. (PDF 136 kb)

Additional file 2: FLAGSHIP investigators. (PDF 8 kb)

## Abbreviations

AMI: Acute myocardial infarction
BMI: Body mass index
GDS5: 5-item Geriatric Depression Scale
GS: Grip strength
HF: Heart failure
HFpEF: Heart failure with preserved ejection fraction
HFrEF: Heart failure with reduced ejection fraction
MMSE: Mini-Mental State Examination
PMADL-8: Performance Measure of Activity in Daily Living-8
SNAQ: Simplified Nutritional Appetite Scale
UWS: Usual walking speed
