# Supplementary material for: A multicenter prospective cohort study to develop frailty-based prognostic criteria in heart failure patients (FLAGSHIP): rationale and design
Source: BMC Cardiovasc Disord. 2018 Aug 2;18:159. doi: 10.1186/s12872-018-0897-y (PMC6090927; doi:10.1186/s12872-018-0897-y)
Supplement: Supplementary file 1 — Hospitals and Ethics Committees enrolled in the FLAGSHIP study. (PDF 136 kb) [file 12872_2018_897_MOESM1_ESM.pdf]

Hospital	Review Board	Approval number (if provided)
Horakata Kohsai Hospital	Ethics Committee of the Hirakata Kohsai Hospital	
Gifu Heart Center	Ethics Committee of Gifu Heart Center	
Kitano Hospital	Kitano Hospital Medical Research Institute Ethics Committee	P16-06-013
Nagoya Tokushukai General Hospital	Tokushukai Group Ethical Committee	TGE00497-016
Nagoya Heart Center	Ethics Committee of Nagoya Heart Center	
Aichi Medical University	Ethics Committee , Aichi Medical University School of Medicine	15-036
Toyohashi Heart Center	Ethics Committee of Toyohashi Heart Center	160203
Kainan Hospital	Ethics Committee of Kainan Hospital	270619-03
Hoshi General Hospital	Ethics Committee of Hoshi General Hospital	43217
New Tokyo Hospital	Ethics Committee of New Tokyo Hospital	
Gifu University Hospital	Ethical Committee of Gifu University School of Medicine	27-138
Mie University Hospital	Human Studies Subcommittee of Mie University Graduate School of Medicine	2924
Nagoya University Hospital	Ethics committee of Nagoya University School of Medicine	2014-0421
Toyota Memorial Hospital	Ethics and Privacy Protection Committee of Toyota Memorial Hospital	
Chubu Rosai Hospital	Ethics Committee of Chubu Rosai Hospital	
Nakatsugawa Municipal Hospital	Ethics Committee of Nakatsugawa Municipal Hospital	
Ohta Nishinouchi Hospital	Ethics Committee of Ohta General Hospital	12
St. Marianna University School of Medicine Yokohama Seibu Hospital	Institutional Review Board of St. Marianna University of School of Medicine	3098
Kyushu Hospital	Committee on Ethics of Clinical Research, Japan Community Healthcare Organization Kyushu Hospital	397
St. Marianna University School of Medicine Toyoko Hospital	Institutional Review Board of St. Marianna University of School of Medicine	3098
Miyakonojo Medical Association Hospital	Ethics Committee of Miyakonojo Medical Association Hospital	27-1
Inazawa Municipal Hospital	Ethics Committee of Inazawa Municipal Hospital	2
Tosei General Hospital	Institutional review board of Tosei General Hospital	512
Fujita Health University Banbuntane Hotokukai Hospital	Fujita Health University Ethical Review Board for Epidemiological and Clinical Studies	15−259
Hokko Memorial Hospital	Ethics Committee of Hokko Memorial Hospital	28-1
Kobe Century Memorial Hospital	Ethics Committee of Kobe Century Memorial Hospital	
Chikamori Hospital	Ethics Committee of Chikamori Hospital	173
St. Marianna School of Medicine Hospital	Institutional Review Board of St. Marianna University of School of Medicine	3098
Nagoya Ekisaikai Hospital	Human (ethics) subjects committee of Ekisaikai Hospital	2016-055
Ogaki Municipal Hospital	Ethics Committee of Ogaki Municipal	20160128-4
