# Supplementary material for: A multicenter prospective cohort study to develop frailty-based prognostic criteria in heart failure patients (FLAGSHIP): rationale and design
Source: BMC Cardiovasc Disord. 2018 Aug 2;18:159. doi: 10.1186/s12872-018-0897-y (PMC6090927; doi:10.1186/s12872-018-0897-y)
Supplement: Supplementary file 2 — FLAGSHIP investigators. (PDF 8 kb) [file 12872_2018_897_MOESM2_ESM.pdf]

FLAGSHIP investigators

Principal investigator: Sumio Yamada (Nagoya University Graduate School of Medicine)

Co-investigators: Hideo Izawa (Fujita Health University Banbuntane Hotokukai Hospital), Toyoaki Murohara (Nagoya University Graduate School of Medicine), Takaaki Kondo (Nagoya University Graduate School of Medicine), Horoki Matsui (The University of Tokyo), Takuji Adachi (Nagoya University Graduate School of Medicine)

Collaborating hospitals and researchers:

Iwatsu K, Fujita R (Hirakata Kohsai Hospital)

Kamisaka K, Nakane E (Kitano Hospital)

Sakui D, Kawamura I (Gifu Heart Center)

Shibata K, Ehara M (Nagoya Heart Center)

Otake H, Shimozato T (Nagoya Tokushukai Hospital)

Abe T, Mizuno T (Aichi Medical University Hospital)

Iida Y, Yamada T (Kainan Hospital)

Nagao T, Sakamoto K (Hoshi General Hospital)

Ando T, Nishigaki K (Gifu University Hospital)

Iritani N, Terashima M (Toyohashi Heart Center)

Ito T, Fujimoto N (Mie University Hospital)

Soga T, Hayashi K (Nakatsugawa Municipal Hospital)

Wakita T, Ishida R (Toyota Memorial Hospital)

Kobayashi K, Okumura T (Nagoya University Hospital)

Uchiyama S, Nishi (New Tokyo Hospital)

Sasamoto Y, Endo N (Ohta Nishinouchi Hospital)

Hasegawa T, Harada K (Chubu Rosai Hospital)

Sato N, Origuchi H (Kyushu Hospital)

Hanada S, Iwakiri H (Miyakonojo Medical Association Hospital)

Kasahara Y, Omiya K (St Marianna University School of Medicine Yokohama City Seibu Hospital)

Kono Y, Izawa H (Fijita Health University Banbuntane Hotokukai Hospital)

Yagi M, Osada N (St Mariannna University School of Medicine Toyoko Hospital)

Takeichi N, Kida K (St Mariannna University School of Medicine Hospital)

Hirasawa J, Kanbara T (Tosei General Hospital)

Ogawa Y, Ishihara T (Inazawa Municipal Hospital)

Kondo K, Fukukawa F (Hokko Memorial Hospital)

Uemura M, Mizutani K (Kobe Century Memorial Hospital)

Tsunekawa Y, Tanimura D (Nagoya Ekisaikai Hospital)

Ban N, Tsuboi H (Ogaki Municipal Hospital)

Maeda H, Kawai K (Chikamori Hospital)
